# Effect of alternative oxidase (AOX) expression on mouse cerebral mitochondria bioenergetics

**DOI:** 10.1016/j.redox.2024.103378

**Published:** 2024-10-01

**Authors:** Belem Yoval-Sánchez, Ivan Guerrero, Fariha Ansari, Zoya Niatsetskaya, Max Siragusa, Jordi Magrane, Vadim Ten, Csaba Konrad, Marten Szibor, Alexander Galkin

**Affiliations:** a Feil Family Brain and Mind Research Institute, Weill Cornell Medicine, 407 East 61st Street, New York, NY, 10065, USA; b Departments of Pediatrics, Robert Wood Johnson Medical School, Rutgers University, New Brunswick, NJ, 08903, USA; c Department of Cardiothoracic Surgery, Center for Sepsis Control and Care (CSCC), Jena University Hospital, 07747, Jena, Germany; d Faculty of Medicine and Health Technology, 33014, Tampere University, Finland

**Keywords:** Alternative quinol oxidase, Mitochondria, Complex I, ROS generation, Reverse electron transfer, Mitochondrial membrane potential

## Abstract

Alternative oxidase (AOX) is an enzyme that transfers electrons from reduced quinone directly to oxygen without proton translocation. When AOX from *Ciona intestinalis* is xenotopically expressed in mice, it can substitute the combined electron-transferring activity of mitochondrial complexes III/IV. Here, we used brain mitochondria from AOX-expressing mice with such a chimeric respiratory chain to study respiratory control bioenergetic mechanisms.

AOX expression did not compromise the function of the mammalian respiratory chain at physiological conditions, however the complex IV inhibitor cyanide only partially blocked respiration by AOX-containing mitochondria. The relative fraction of cyanide-insensitive respiration increased at lower temperatures, indicative of a temperature-controlled attenuation of mammalian respiratory enzyme activity.

As AOX does not translocate protons, the mitochondrial transmembrane potential in AOX-containing mitochondria was more sensitive to cyanide during succinate oxidation than during malate/pyruvate-supported respiration. High concentrations of cyanide fully collapsed membrane potential during oxidation of either succinate or glycerol 3-phosphate, but not during malate/pyruvate-supported respiration. This confirms AOX's electroneutral redox activity and indicates differences in the proton-translocating capacity of dehydrogenases upstream of the ubiquinone pool. Our respiration data refutes previous proposals for quinone partitioning within the supercomplexes of the respiratory chain, instead supporting the concept of a single homogeneous, freely diffusing quinone pool.

Respiration with either succinate or glycerol 3-phosphate promotes reverse electron transfer (RET) towards complex I. AOX expression significantly decreased RET-induced ROS generation, with the effect more pronounced at low temperatures. Inhibitor-sensitivity analysis showed that the AOX-induced decrease in H_2_O_2_ release is due to the lower contribution of complex I to net ROS production during RET.

Overall, our findings provide new insights into the role of temperature as a mechanism to control respiration and highlight the utility of AOX as a genetic tool to characterize both the distinct pathways of oxygen reduction and the role of redox control in RET.

## Highlights

• Functional alternative quinol oxidase (AOX) was expressed in mice.
• AOX presence does not significantly alter routine bioenergetics of mouse brain mitochondria.
• AOX provides cyanide-insensitive mitochondrial respiration.
• Effects of AOX are more pronounced at lower temperatures.
• AOX decreases ROS production by mitochondrial complex I during reverse electron transfer.

## 1 Introduction

Mitochondria play crucial roles in ATP synthesis, maintenance of redox equilibrium, and cellular signaling pathways. Manifestation of mitochondrial malfunctions contributes significantly to various pathological human conditions, inherited [1] and acquired, including cardiovascular and neurodegenerative diseases [2,3], metabolic disorders [4,5], acute stress response [6], cancer development [7], and immune cell reprogramming [8].

Aerobic mammalian cells rely mostly on ATP production via mitochondrial oxidative phosphorylation based on the combined activity of enzymes of catabolism, the respiratory chain, and ATP synthase. The process begins with the transfer of electrons derived from different substrates to oxygen via a system of respiratory chain enzymes located in the inner mitochondrial membrane. A fraction of the energy released in the redox reactions is used by complexes I, III, and IV to pump protons across the inner membrane, thereby forming the proton-motive force, the driving force for ATP production. The backflow of protons from the intermembrane space to the mitochondrial matrix via the ATP-synthase drives the formation of ATP from ADP and inorganic phosphate.

In brain mitochondria, there are at least three entry points for electrons that provide electron flux at high rates: proton-pumping complex I that oxidizes NADH, succinate dehydrogenase of the TCA cycle (SDH or complex II), and glycerol 3-phosphate dehydrogenase (mGPDH) on the outer side of the inner membrane [9]. Ubiquinone is a direct electron acceptor for all of these dehydrogenases, and further electron flux through the respiratory chain is controlled by the rates of electron input (reduction) and output (oxidation) at the level of the ubiquinone pool. The only enzyme that oxidizes ubiquinol in the inner membrane is complex III (*bc*_1_ complex), which transfers electrons downstream to complex IV, the only terminal oxidase in mammals that uses oxygen as a final electron acceptor. If the electron transfer from ubiquinol is blocked by genetic defects or specific inhibitors, such as antimycin A for complex III or cyanide for complex IV, respiration is impaired, proton pumping is halted, and consequently ATP synthesis and all upstream metabolic pathways are blocked. Studies of bacterial and plant cyanide-resistant respiration resulted in the discovery of the alternative terminal quinol oxidase (AOX), which transfers electrons from ubiquinol directly to oxygen [10]. This enzyme is absent in mammals and can substitute for the combined electron-transferring activity of complexes III and IV if xenotopically expressed. Since AOX does not pump protons across the inner membrane, its activity does not directly add to the proton-motive force but instead dissipates the redox energy of the quinol/oxygen span (Δ*E*^0^∼800 mV) as heat. Construction of chimeric respiratory chains via xenotopic expression of AOX in mammals [11,12] is not only an efficient tool to study mitochondrial bioenergetics but also offers a possibility to modulate respiration to ameliorate various mitochondria-related pathologies.

The aim of this study was to compare the bioenergetics of brain mitochondria from non-transgenic (nTG) mice with canonical respiratory chain and AOX-expressing transgenic (TG) mice. We showed the impact of AOX expression in mitochondria capable of reverse electron transfer (RET) using two different routes of electron supply, *i.e.* oxidation of succinate and glycerol 3-phosphate. We assessed the effect of AOX on classical parameters of intact mitochondria preparation such as respiration, cyanide-sensitivity, generation of membrane potential across the inner mitochondrial membrane, and ROS production in a temperature-dependent manner.

## 2 Materials and Methods

### 2.1 Mouse models

The animal protocol was approved by the Institutional Animal Care and Use Committee of Weill Cornell Medicine. All experiments were conducted in accordance with the Guidelines for the Care and Use of Laboratory Animals of the National Institutes of Health and the ARRIVE guidelines. We obtained ubiquitous expression of AOX from *Ciona intestinalis* by crossing Rosa26-CAG-SNAPf-AOX mice [13] (hereafter called AOX mice) with mice expressing CRE recombinase directed by the ubiquitous human β-actin gene promoter (B6.FVB-Tmem163^Tg(ACTB-cre)2Mrt^/EmsJ; The Jackson Laboratories, 033984). Offspring was genotyped by PCR and efficiency of Cre-recombination was confirmed by the presence of cyanide-resistant respiration. Heterozygous AOX mice develop normally and exhibit normal physiology without any deleterious consequences under normal physiological conditions. All mice were kept on a C57BL/6J background.

### 2.2 Mitochondria isolation

To isolate intact mitochondria, a modified protocol combining differential centrifugation and digitonin treatment was used [14]. All operations were performed on ice or at 4 °C. The forebrain hemispheres were excised and immediately placed into an ice-cold MSE isolation medium (225 mM mannitol, 75 mM sucrose, 20 mM HEPES-Tris (pH 7.4), 1 mM EGTA, 1 mg/ml BSA). Tissue was homogenized with 40 strokes by a Dounce homogenizer (tight pestle “B”) in 10 ml of the MSE, diluted two-fold by the same medium, and centrifuged at 4000×*g* for 4 min. Digitonin (0.02 %) was added to the supernatant, and centrifuged again at 10,000×*g* for 10 min, the pellets were resuspended in MSE without BSA, and washed twice. The final mitochondrial pellet was resuspended in 0.1 ml of the same medium, stored on ice and used within 3–4 h after isolation. Protein was quantified using BCA assay (Pierce).

### 2.3 Western blot

Tissue homogenates or isolated mitochondria fractions were analyzed by western blot, as described before [15]. To detect AOX, rabbit polyclonal AOX antiserum was used [12], (21st Century Biochemicals, custom made, dilution 1:50,000; 2 % milk, 1h at room temperature). Horseradish peroxidase-conjugated secondary goat anti-rabbit Ab were used for chemiluminescence detection (Jackson Immunoresearch; catalog no. 111-035-144: dilution 1:10,000; 2 % milk 1h at room temperature). Images of the blots were obtained using FluorChem M Western Blot Imaging System (Protein Simple).

### 2.4 Respiration, H_2_O_2_ release, and membrane potential measurements in intact brain mitochondria

Oxygen consumption, the release of H_2_O_2,_ and membrane potential were measured using a high-resolution respirometer (O2k oxygraph, Oroboros Instruments, Innsbruck, Austria) equipped with two-channel optical setup to monitor fluorescence (excitation/emission 525/580 nm) [14]. Mitochondria were stored on ice and added to the oxygraphy chamber to the final concentration of 0.1 mg/ml of protein. Chamber contained 2 ml measuring buffer composed of 125 mM KCl, 0.2 mM EGTA, 20 mM HEPES-Tris, 4 mM KH_2_PO_4_, pH 7.4, 2 mM MgCl_2_, 1 mg/ml BSA and supplemented with specific substrates. The following substrates were used: 2 mM malate and 5 mM pyruvate for complex I-supported respiration), or 5 mM succinate and 1 mM glutamate (for complex II), and 40 mM glycerol 3-phosphate (for mGPDH). After 3–5 min of recording oxygen consumption rate in non-phosphorylating conditions, 200–400 μM ADP was added to initiate State 3 (phosphorylating) respiration. If H_2_O_2_ release was simultaneously assessed in the same assay measuring buffer was supplemented with 10 μM Amplex UltraRed (Invitrogen), 4 U/ml horseradish peroxidase, and 5 U/ml superoxide dismutase. The raw fluorescence resorufin fluorescence was calibrated by addition of H_2_O_2_ aliquots of known concentration (250 nM). If membrane potential was assessed, 1 μM safranin O was added instead of H_2_O_2_ detection system and fluorescence was measured as described in Ref. [16]. At the end of the experiment, maximum fluorescence was recorded after addition of 40 μg/ml alamethicin or 1 μM FCCP to fully collapse membrane potential.

Measurements were performed at 25 °C, unless specified otherwise. When temperature-dependence was assessed, the Oroboros electrode was calibrated for each temperature and mitochondria were added to the measuring buffer with substrates at 15, 25 or 37 °C. Total time the measurements was around 10–15 min. Respiration rates and fluorescence emission data were recorded using the DatLab software (version 6.1.0.7) at 1Hz time resolution and analyzed in Microcal Origin. Oxygen consumption in nmol O_2_/min or H_2_O_2_ release in nmol H_2_O_2_/min was normalized by the amount of protein in mg added to the buffer. All results are expressed as means ± SD for at least three biological replicates unless stated otherwise.

### 2.5 List of reagents

Chemicals were purchased from Sigma: adenosine 5-diphosphate potassium salt (ADP) (#A5285), antimycin A (#A8674), bovine serum albumin (BSA) essentially fatty acid free (#A6003), digitonin (#D141), EGTA (#E3889), glycerol 3-phosphate (#61668), glutamate (# 49449), FCCP (#C2920), HEPES (# 4034), H_2_O_2_ (#216763), horseradish peroxidase (#P8375), KCl (#P3911), malate (#233935), mannitol (#63559), rotenone (#R8875), potassium cyanide (#207810), SOD (#S9697), sucrose (#S7903), pyruvate (#P5280), safranin O (#S8884), and succinate (#14160). Alamethicin was purchased from Cayman Chemical (#11425). Amplex UltraRed (#A36006) and Pierce BCA protein assay kit (#23225) were from Thermo Fisher Scientific. Precision Plus Protein Dual Color Standards (#1610374) were from Bio-Rad.

## 3 Results

1. AOX expression provides cyanide-resistant respiration.

**Fig. 1:**
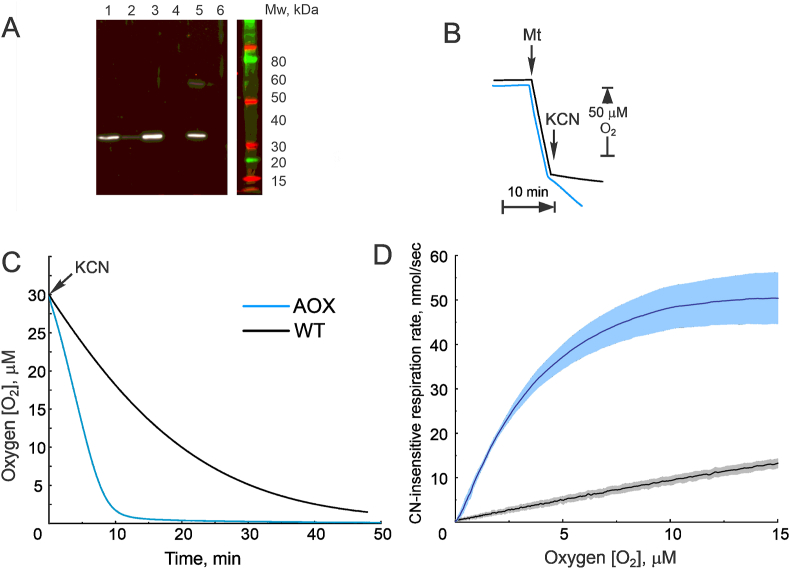
*AOX expression provides cyanide-resistant respiration in the brain mitochondria.* (A) Representative western blot image with antiserum against AOX using 20 μg of protein homogenate from lung (1), kidney (2), and brain (3) of AOX-expressing mice or brain of nTG animals (4). Lanes 5 and 6 are brain mitochondria (10 μg protein per well) from AOX or nTG mice, respectively. (B) Representative trace of State 3 (phosphorylating) succinate-supported respiration of nTG (black) and AOX-containing (blue) intact brain mitochondria. Reaction was started by the addition of mitochondria (0.05 mg/ml) to a standard media supplemented with substrates and 0.5 mM ADP and then respiration was measured at 37 °C as described in the Materials and Methods. Addition of mitochondria (Mt) or 0.1 mM cyanide (KCN) is shown by arrows. (C) Representative trace of succinate-supported mitochondrial State 3 respiration after addition of 50 μM cyanide. Mitochondria from nTG or AOX-expressing mice (0.1 mg/ml, black and blue traces respectively) were incubated in the presence of succinate and 0.5 mM ADP until oxygen reached 30 μM, and then cyanide was added as shown by arrow (KCN). (D) Oxygen dependence of cyanide-insensitive respiratory rates of mitochondria isolated from nTG and AOX mice (black and blue traces, respectively) calculated from experiments shown in panel C. Error bars represent one standard deviation based on triplicate measurements. (For interpretation of the references to colour in this figure legend, the reader is referred to the Web version of this article.)

First, the presence of AOX in brain tissue homogenates or in isolated intact mitochondria from transgenic mice was confirmed by Western blot and by functional activity measurements (Fig. 1A and B). Clear bands in the region of 35 kDa corresponding to AOX molecular weight were only detected in the tissue homogenates or isolated mitochondria samples from AOX-expressing animals (Fig. 1A; lanes 1–3 and 5). Succinate-supported respiration of intact brain mitochondria from nTG mice was fully inhibited by cyanide. Mitochondria from AOX animals catalyze direct oxidation of quinol by oxygen, and therefore their respiration was only partially inhibited by cyanide (Fig. 1B, blue trace). The rates of cyanide-resistant oxygen consumption were then measured at low levels of oxygen as shown in Fig. 1C. Oxygen consumption rate of mitochondria with canonical respiratory chain displayed linear dependence on oxygen (Fig. 1D, black trace), unlike the reaction of AOX-containing mitochondria where classical Michaelis Menten hyperbolic dependence with *K*_*m*_ of 3.0 ± 0.2 μM for O_2_ was observed (Fig. 1D, blue trace). This confirms that a specific cyanide-insensitive enzyme, with lower than complex IV affinity to oxygen catalyzes oxygen consumption in AOX-expressing mice. Therefore, AOX is present in the functionally active state in the intact mitochondria prepared from the brain of transgenic mice.

2. Effect of temperature on respiration.

**Fig. 2:**
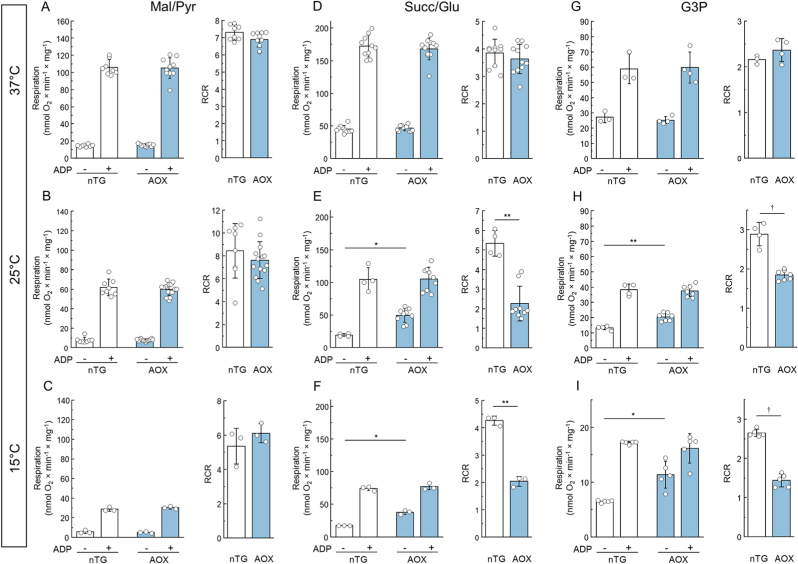
*Effect of temperature on State 2, State 3 respiration and Respiratory Control Ratio (RCR) of intact brain mitochondria isolated from nTG and AOX-containing mice (white and blue bars, respectively).* Three types of substrates were used: 1 mM malate and 5 mM pyruvate for complex I-supported respiration (A–C), 5 mM succinate and 2 mM glutamate for complex II-based respiration (D–F), and 40 mM glycerol 3-phosphate for mGPDH (G–I). Reaction started with 0.1 mg/ml mitochondria and measured as described in the Materials and Methods section at 37, 25 and 15 °C (upper, middle, and bottom panels, respectively). 400 μM ADP was used for stimulation of State 3 respiration. Values are shown as mean ± SD (n = 3–10, ∗p < 0.001, ∗∗p < 0.0001, ^†^p < 0.00001, *t*-test). (For interpretation of the references to colour in this figure legend, the reader is referred to the Web version of this article.)

Next, we tested the effect of AOX presence on the oxidation of different substrates by intact brain mitochondria at different temperatures. At 37 °C with any substrate, the presence of AOX did not affect the rate of State 2 (non-phosphorylating) or State 3 (phosphorylating) respiration, and, therefore, the respiratory control ratio (RCR) (Fig. 2A–D, G). However, at lower temperatures non-phosphorylating State 2 respiration was higher in mitochondria from AOX-expressing mice, compared to nTG control mice, indicating lesser mitochondria coupling when AOX is present (Fig. 2E, F, H, I). Note that this effect was only observed using succinate or glycerol 3-phosphate as substrates.

3. Temperature-dependence of cyanide sensitivity on mitochondrial respiration.

**Fig. 3:**
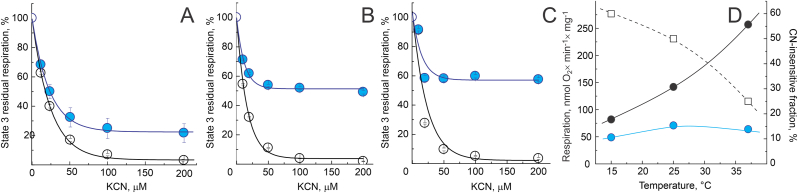
*Cyanide sensitivity of intact brain mitochondria respiration at different temperatures.* Succinate-supported State 3 phosphorylating respiration was assessed at 37, 25 and 15 °C (panels A, B, and C respectively) as described in Materials and Methods using 0.1 mg/ml of mitochondria from nTG (black) or AOX-expressing (blue) mice. (D) Values of State 3 respiration of AOX-containing mitochondria are plotted in the absence and the presence of 0.2 mM cyanide (black and blue circles, respectively). Ratio of these two values, *i.e.*, fraction of cyanide insensitive respiration at different temperatures is shown as squares (percentage, right axis). (For interpretation of the references to colour in this figure legend, the reader is referred to the Web version of this article.)

To investigate the contribution of AOX to cyanide-resistance we performed careful inhibitor titration of State 3 phosphorylating respiration of mitochondria from nTG and AOX-expressing mice at different temperatures (Fig. 3). As in Fig. 1B, concentrations of cyanide above 0.1 mM completely inhibited respiration of mitochondria from nTG mice, but not of the AOX-containing respiratory chain. The residual cyanide-resistant fraction of overall respiration mediated by AOX increased significantly when temperature was lowered (Fig. 3A–C). Considering the difference in body temperature of the warm-blooded mouse and the poikilothermic *Ciona intestinalis*, it stands to reason that the lower contribution of AOX to cyanide-resistance at higher temperature is unlikely the effect of a decrease of AOX-catalyzed reaction rate, but rather due to the greater contribution of mammalian components of such chimeric respiratory chain to the overall respiration. Indeed, Fig. 3D demonstrates that the dramatic increase of relative fraction of cyanide-insensitive respiration with temperature decrease was due to the decline of total State 3 respiration. The absolute rate of AOX-catalyzed respiration was only slightly affected by the temperature (Fig. 3D, blue circles).

4. Effect of AOX expression on maintenance of mitochondrial membrane potential.

**Fig. 4:**
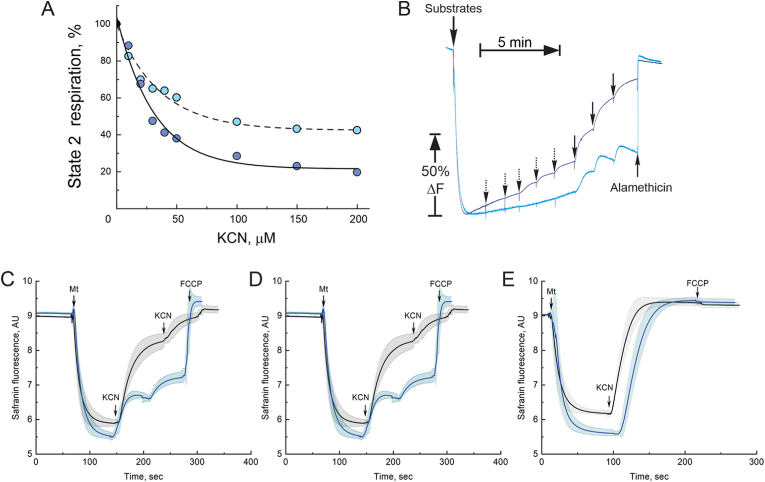
*Effect of AOX expression on mitochondrial membrane potential.* (A) Cyanide titration of AOX-containing mitochondria oxidizing succinate or malate/pyruvate couple in non-phosphorylating State 2 (dark and light blue circles, respectively). State 2 respiration was assessed at 25 °C with 0.1 mg/ml mitochondria as described in Materials and Methods. 100 % corresponds to 52 ± 6 and 9 ± 1 nmol O_2_ × min^−1^ × mg protein^−1^ for succinate- or malate/pyruvate-supported respiration. (B) Representative trace of the experiment assessing the effect of cyanide on membrane potential measured as safranin fluorescence using Oroboros respirometer fluorescent setup in AOX-containing mitochondria oxidizing succinate or malate/pyruvate in non-phosphorylating State 2 (dark and light blue traces, respectively). Dashed and solid arrows indicate addition of 10 μM or 50 μM KCN, respectively. 40 μg/ml of alamethicin was added at the end of the experiment to fully dissipate the membrane potential. (C–E) Mitochondrial membrane potential assessment in mitochondria from nTG (black) or AOX-expressing (blue) mitochondria respiring on malate/pyruvate (C), succinate (D) or glycerol 3-phosphate (E). Safranin fluorescence was assessed at 25 °C as described in Materials and Methods and arrows indicate addition of 0.1 mg/ml intact mitochondria (Mt), 4 mM cyanide (KCN) or 1 μM FCCP as uncoupler (FCCP). Error bars represent one standard deviation based on triplicate measurements. (For interpretation of the references to colour in this figure legend, the reader is referred to the Web version of this article.)

We were specifically interested in characterizing the effect of AOX presence on the mitochondrial membrane potential. First, using increasing doses of KCN, we established that the cyanide insensitive fraction of State 2 (non-phosphorylating) respiration in AOX-containing mitochondria was significantly higher with malate/pyruvate than succinate/glutamate as substrates (Fig. 4A). We further confirmed these observations by using a fluorescent indicator of membrane potential, safranin O. When complex IV was gradually inhibited by increasing concentrations of cyanide, mitochondrial membrane potential decreased (Fig. 4B). However, with succinate-supported respiration, sequential addition of cyanide resulted in a much more depolarized potential range, nearing to complete dissipation (Fig. 4B, dark blue trace). Oppositely, when malate and pyruvate were used as substrates, potential was only slightly affected by increasing cyanide concentration and never completely dissipated (Fig. 4B, light blue trace). Therefore, the capacity to generate mitochondrial membrane potential in the presence of cyanide during State 2 respiration was higher if a combination of malate/pyruvate was used. This observed difference in the response to cyanide for two respiratory substrates is due to the ability of complex I to translocate protons across the membrane and the absence of proton pumping in complex II. This interpretation was further confirmed by comparison of safranin fluorescence response to full inhibition of complex IV in nTG and AOX-containing mitochondria oxidizing three different substrates: malate and pyruvate, succinate, or glycerol 3-phosphate (Fig. 4C–E). As expected, addition of cyanide resulted in complete dissipation of the membrane potential in the canonical respiratory chain due to inhibition of complex IV, regardless of substrate used. In mitochondria from transgenic mice, no potential across the membrane could be generated after the addition of cyanide when complex II or mGPDH were working together with AOX (Fig. 4C–E, blue traces). The only condition when membrane potential could be maintained in the presence of cyanide was when AOX-containing mitochondria oxidized malate and pyruvate. This respiratory activity resulted in proton-translocating at complex I and in the maintenance of mitochondrial potential (Fig. 4C).

5. Effect of AOX expression on ROS production

**Fig. 5:**
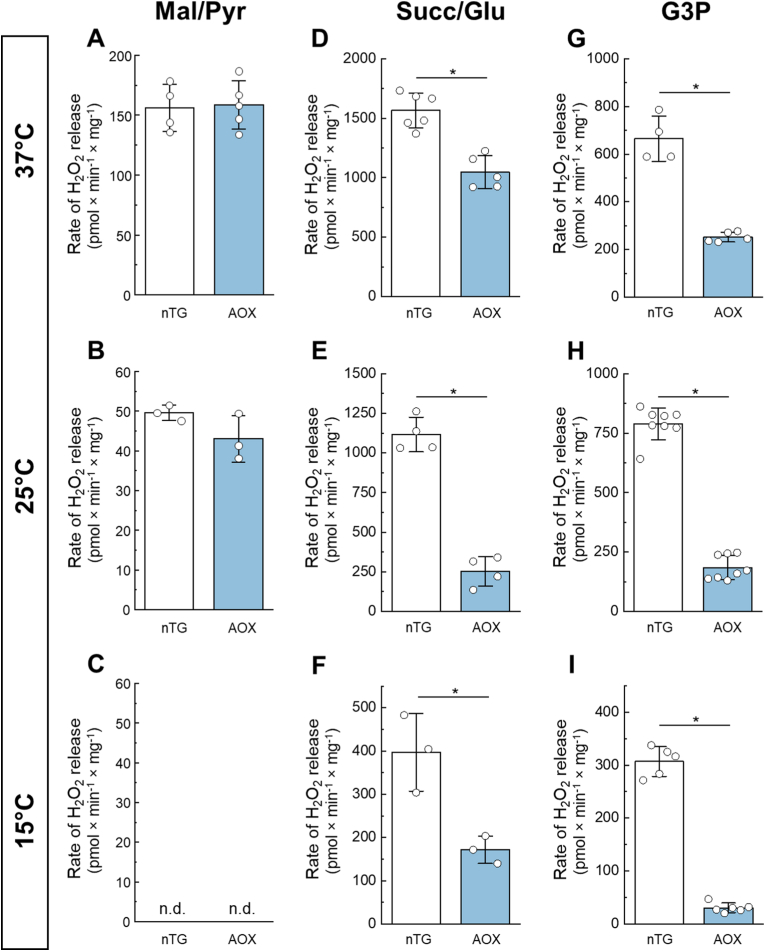
*ROS generation by brain mitochondria from nTG and AOX-expressing mice respiring on different substrates*. H_2_O_2_ release was measured as described in Materials and Methods in the standard assay using various substrates oxidized in non-phosphorylating State 2 at different temperatures. The following substrate combinations were used malate/pyruvate (A–C), succinate/glutamate (E–F), and glycerol 3-phosphate (G–I) at temperatures 37 °C (upper row, A, D, G), 25 °C (middle row, B, E, H) and 15 °C (bottom row, C, F, I). No detectable H_2_O_2_ release was measured with malate/pyruvate at 15 °C. Values are shown as mean ± SD (n = 3–10, ∗p < 0.001, *t*-test).

We next investigated the effect of AOX expression on ROS generation by intact mitochondria. We used a fluorescent assay with Amplex UltraRed and horseradish peroxidase for detecting the emission of H_2_O_2_ during respiration in intact mitochondria using different substrates. The cumulative results are shown in Fig. 5. As we [[16], [17], [18]] and others [[19], [20], [21], [22], [23], [24]] demonstrated before, the highest rate of the H_2_O_2_ release by the brain mitochondria is found during oxidation of succinate or glycerol 3-phosphate in conditions of reverse electron transfer (RET). When these substrates are oxidized by canonical respiratory chain in non-phosphorylating conditions, combined proton-pumping activity of complexes III and IV provides proton-motive force which is sufficient to push a fraction of electrons from quinol pool upstream toward complex I to reduce the matrix NAD^+^ pool. This results in reduction of complex I redox centers and increases the rate of one-electron reduction of oxygen, generating superoxide, predominantly at the reduced or semi-reduced flavin of complex I [[25], [26], [27], [28]] (see however [[29], [30], [31]] for a different interpretation). High proton-motive force and quinol/quinone ratio are required to direct electrons via RET to complex I. Therefore, in the presence of AOX that can oxidize the quinol pool without proton translocation, we expected lower values of H_2_O_2_ emission from intact mitochondria oxidizing RET-supporting substrates succinate or glycerol 3-phosphate. Confirming earlier observations [12,32,33], we demonstrated a strong diminishing effect of AOX on ROS generation only when RET substrates were used (Fig. 5, succinate and glycerol 3-phosphate data). AOX effect on ROS generation in conditions of RET was more pronounced at lower temperatures (see also respiration data in Fig. 2). There was no significant effect of AOX on ROS generation when malate/pyruvate couple was used and no detectable H_2_O_2_ release was observed at 15 °C with these substrates.

**Fig. 6:**
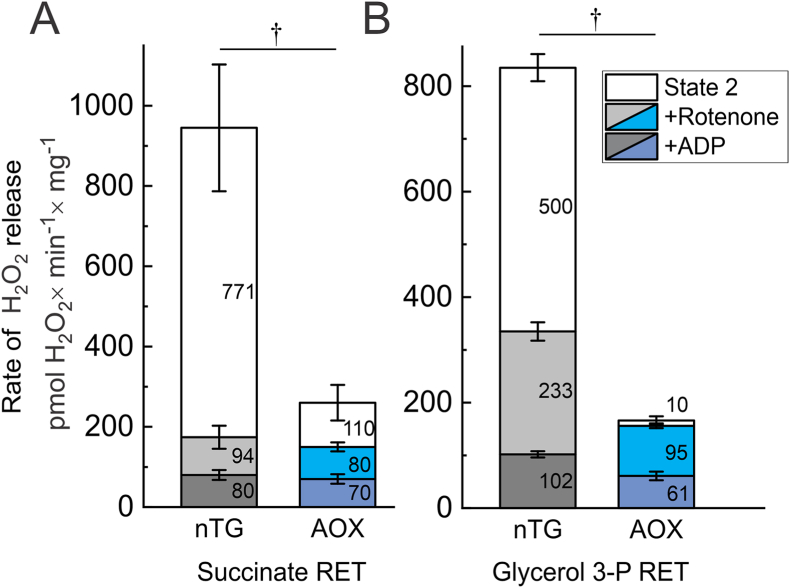
*Effect of rotenone on RET-stimulated ROS generation*. H_2_O_2_ release during State 2 succinate- or glycerol 3-phosphate-supported respiration (A and B, respectively). H_2_O_2_ release was measured as described in Materials and Methods section using standard assay conditions with 0.1 mg/ml of mitochondria from nTG and AOX-expressing mice (grey and blue bars respectively) at 25 °C. After recording of H_2_O_2_ release during state 2 respiration, 2 μM rotenone was added to block electron transfer at complex I and then 400 μM ADP was added to initiate State 3 respiration and decrease membrane potential. Numbers indicate mean values of H_2_O_2_ release in pmol H_2_O_2_ × min^−1^ × mg^−1^ in corresponding conditions. Values are shown as mean ± SD (n ≥ 4, ^†^p < 0.00001, *t*-test). (For interpretation of the references to colour in this figure legend, the reader is referred to the Web version of this article.)

To further confirm our interpretation of the AOX effect on ROS generation by intact mitochondria in RET conditions we tested the effect of complex I-specific quinone-like inhibitor rotenone on H_2_O_2_ release during oxidation of succinate or glycerol 3-phosphate during State 2 respiration as shown in Fig. 6. Net H_2_O_2_ release during succinate-supported State 2 respiration was around 30 % in the AOX-containing mitochondria without the inhibitor (total height of the bar graphs at Fig. 6A). After addition of rotenone to block electron transfer in complex I we were able to estimate the contribution of that enzyme to the net H_2_O_2_ release. With succinate, in the canonical respiratory chain, almost 80 % of total H_2_O_2_ release (771 ± 158 pmol H_2_O_2_ × min^−1^ × mg protein^−1^) was eliminated by rotenone, indicating that under conditions of RET the main ROS-generating site(s) are located upstream of the quinone-binding site of complex I. In AOX-containing mitochondria rotenone addition decreased total H_2_O_2_ release by 40 % giving a calculated contribution of complex I value of 110 ± 45 pmol H_2_O_2_ × min^−1^ × mg protein^−1^. Remarkably, the level of H_2_O_2_ emission in the presence of rotenone was almost indistinguishable for both types of mitochondria. The decrease of membrane potential after adding ADP further decreases the H_2_O_2_ release, probably reducing ROS generation at complex II and mGDPH. Qualitatively similar effects were observed for glycerol 3-phosphate-supported RET, where the presence of AOX almost completely abolished the potential dependent fraction of H_2_O_2_ release (Fig. 6B). This suggests the absence of complex I contribution to the overall H_2_O_2_ release in AOX-containing mitochondria when oxidizing glycerol 3-phosphate. Our results indicate that the AOX-induced decrease in H_2_O_2_ release is due to a reduction in RET-stimulated ROS formation upstream of the quinone-binding site of complex I.

## 4 Discussion

AOX is an enzyme expressed in plants, fungi, some protozoans, and metazoans including sea tunicates [10,[34], [35], [36]] but absent in vertebrates. Mitochondrial-specific expression of AOX has been reported for human cell lines [37], flies [38], and mice [11,13] and was suggested to be used as a tool to study mechanisms of mitochondrial bioenergetics. AOX can function as a rescue mechanism to protect the cell from deleterious effects caused by disruptions in the respiratory chain downstream of the quinone junction. It can also serve as a tool to test disease paradigms and potentially have therapeutic applications [39,40]. In this study, we characterized in detail the consequences of the expression of AOX from *Ciona intestinalis* in mice on the bioenergetics of brain mitochondria.

Here, we show that AOX expression has almost no effect on overall respiration rate in State 2 or 3 at physiologically relevant temperature, but slightly decreases respiratory control at lower temperatures with non-NADH substrates such as succinate or glycerol 3-phosphate. This is in agreement with the temperature effect on the coupling of succinate-supported respiration observed in liver mitochondria [41,42]. AOX provides cyanide-insensitive respiration in mitochondria with kinetic parameters different from cytochrome *c* oxidase. Our results demonstrate that cyanide-insensitive respiration in AOX mitochondria manifests classical Michaelis-Menten kinetics in relation to oxygen with apparent *K*_*m*_ for oxygen of 3.0 μM, which is at least one order of magnitude higher than that of complex IV [43,44]. A similar range of oxygen affinity was demonstrated for AOX expressed in pulmonary arterial smooth muscle cells [32]. There is no available data on oxygen affinity for AOX from *C. intestinalis*; however, the *K*_*m*_ for oxygen varies depending on the species and has been reported in the range of 1–5 μM for the enzymes from the parasitic microorganism *T. brucei* and various plants [45]. Our evidence that AOX has a lower affinity to oxygen, compared to complex IV suggests, that *in situ*, the contribution of AOX to overall respiration might be defined by the level of oxygen as well as the quinol/quinone ratio. At the same time, linear dependence of oxygen consumption on oxygen concentration in the absence of AOX in mitochondria from nTG mice probably reflects the second-order reaction of ROS generation by the mitochondrial respiratory chain observed by us [17,18] and others [27,46].

We were unable to detect any differences in respiration between mitochondria from nTG and AOX-expressing mice under physiological temperatures, confirming earlier observations for AOX-containing heart mitochondria [12]. However, at lower temperatures, expression of AOX resulted in higher State 2 non-phosphorylating respiration and therefore lower respiratory control ratio. Together with the increase of the cyanide-insensitive fraction of respiration in AOX-containing mitochondria with temperature decrease, this indicates a higher relative electron flux via AOX at lower temperatures. Total respiration flux increases with temperature, but the cyanide-insensitive part does not change significantly, therefore the percentage of AOX-dependent cyanide-insensitive respiration decreases. Interestingly, the reported optimal temperature for *Ciona intestinalis* is 10–20 °C [47] however, we did not detect any significant difference in AOX activity (measured as cyanide-resistant respiration) at the interval between 15 and 37 °C. The fact that the AOX-catalyzed reaction is not inhibited at the higher temperature underlines its value as a promising therapeutic suitable for use in human pathologies.

We found that the cyanide-insensitive fraction of respiration also depends on the substrate and is higher with malate/pyruvate than with succinate/glutamate. An increase in the cyanide-insensitive activity provided by AOX thus could be explained, at least partially, by AOX activation by pyruvate as observed for the plant enzyme [48], (but not for *T. brucei* AOX [45]). This, however, contradicts the fact that the rate of complex II-supported respiration is higher than oxygen consumption with complex I substrates. It also seemed possible that a higher quinol/quinone ratio can be sustained by the presence of membrane potential, since the combined activity of complex I and AOX affords vectorial proton translocation across the membrane. This contrasts with the oxidation of succinate by complex II and AOX where no proton pumping occurs. Indeed, when membrane potential was titrated by cyanide in AOX mitochondria in the presence of either succinate or malate/pyruvate couple, increased concentrations of inhibitor fully dissipate potential only if complex II substrate is used. We further confirmed this by assessing the response of membrane potential to a high bolus of cyanide added to either mitochondria from nTG or AOX-expressing mice oxidizing different substrates. Since cyanide fully inhibits respiration in the canonical respiratory chain, the addition of the inhibitor rapidly collapses potential when nTG mitochondria oxidize any substrate. In AOX-expressing mice when either complex II or mGPDH is working in combination with AOX, oxidation of both substrates is not coupled to proton pumping, and therefore potential is fully collapsed after the addition of cyanide. This does not happen when AOX-containing mitochondria oxidize malate/pyruvate, since in combination with complex I, AOX indirectly allows the generation of membrane potential due to the translocation of 4H^+^ per turnover of complex I. Thus, complex I-AOX-linked respiration provides at least 50 % of the pumping capacity of the canonical respiratory chain [[49], [50], [51], [52]], which appears to suffice to maintain the mitochondrial membrane potential, *i.e.*, driving force for ATP production.

The data reported here, however, do not allow us to determine the degree of AOX participation in overall respiration in the absence of inhibitors of the respiratory chain downstream of the quinone pool. We demonstrated that in non-phosphorylating State 2 conditions complex I-supported respiration demonstrated higher cyanide-resistance than respiration with succinate (*i.e.*, 8 or 4 protons are pumped one atom of oxygen consumed). We also showed the presence of membrane potential in AOX-containing mitochondria only when mitochondria oxidize substrates supporting complex I-based respiration. Interestingly, we did not observe any significant difference in membrane potential between mitochondria from non-transgenic and AOX-expressing mice in the RET condition, when AOX-containing mitochondria produce only half of ROS, confirming earlier observations for the intact heart mitochondria [12,53]. These two observations further strengthen the assumption that it is unlikely that significant electron flux via AOX takes place when complexes III or IV are not inhibited or when mitochondria are depolarized. In support of this notion, a previous study revealed little to no difference between mice ubiquitously expressing AOX and wild-type littermates [11]. However, if mitochondrial membrane potential is approaching the threshold and quinol availability for AOX is high, the enzyme activity is stimulated and AOX relieves electron pressure, decreasing electron flux towards complex I [12,53].

Perhaps the most important observation from this and previous [12,53] studies is the fact that AOX expression significantly affects RET-supported ROS generation by intact brain mitochondria. RET takes place when brain mitochondria oxidize either succinate or glycerol 3-phosphate in “coupled” conditions. Supported by proton-motive force generated by complex III and cytochrome oxidase, a small fraction of electrons from quinol pool is directed upstream, therefore reducing complex I from downstream. In these conditions, the complex I reaction can be reversed and electrons from quinol are transferred upstream towards the FMN-binding site of the enzyme and then further to the available NAD^+^ in the matrix. The amount of intramitochondrial purine nucleotides is limited, therefore after complete reduction of NAD^+^ to NADH, electrons on the flavin could escape to any available donor, *e.g.*, oxygen molecule with the formation of either superoxide [26,[54], [55], [56]] or H_2_O_2_ [57,58]. Steady-state oxidation of succinate or glycerol 3-phosphate RET in intact mitochondria or submitochondrial particles supports the highest rate of ROS generation *in vitro* in comparison with other respiratory substrates [16,[18], [19], [20], [21],^23^,^24^,^59^], or even in the presence of antimycin, known to induce ROS generation in complex III [60,61]. The preparations of mouse brain intact mitochondria routinely used in our laboratory show an average fraction of electrons directed towards ROS production at complex I during succinate or glycerol 3-phosphate supported RET between 3.5 and 5 % of total respiration, depending on the assay conditions. RET is supported by the high quinol/quinone ratio in the membrane and by the presence of proton-motive force. AOX can potentially diminish both of those components by oxidizing quinol without proton pumping, so dissipating redox energy as heat, and decreasing the ROS production. The fact that ROS production was the same for nTG and AOX mitochondria in the presence of rotenone discards the participation of AOX as a producer or scavenger of ROS.

Another, more remote, implication of this study and original reports [11,12], is that despite AOX being found in *Tunicata*, which is a different subphylum from mammals, the enzyme can participate in quinol oxidation in mouse brain mitochondria. Fundamentally, it suggests that quinol produced either by complex I or by complex II is available for AOX-catalyzed reactions, and therefore the quinone pool is non “occluded” within respiratory chain supercomplexes (composed of complexes I, III, and IV) as was suggested in the past (reviewed in Ref. [62]).

In conclusion, xenotopically expressed AOX from *C. intestinalis* is catalytically active in the mouse brain mitochondria and can be served as a tool to pinpoint mechanistic details of mitochondria bioenergetics. Most likely, AOX is stimulated only when the quinone pool is highly reduced by the presence of inhibitors or at high values of membrane potential such as non-phosphorylating conditions. Our analysis suggests a lack of evidence for quinone pool occlusion within respiratory chain supercomplexes and supports the concept of a freely diffusible Q-pool, proposed more than 50 years ago [63]. The main feature of AOX-containing mitochondria is a significant decrease in the electron flux from quinol towards complex I during oxidation of succinate- or glycerol 3-phosphate in RET-like conditions.

## CRediT authorship contribution statement

**Belem Yoval-Sánchez:** Writing – review & editing, Supervision, Investigation, Formal analysis, Data curation, Conceptualization. **Ivan Guerrero:** Writing – review & editing, Methodology, Investigation. **Fariha Ansari:** Methodology, Data curation. **Zoya Niatsetskaya:** Conceptualization. **Max Siragusa:** Writing – review & editing, Methodology. **Jordi Magrane:** Writing – review & editing, Methodology. **Vadim Ten:** Writing – review & editing, Conceptualization. **Csaba Konrad:** Writing – review & editing, Methodology, Data curation. **Marten Szibor:** Writing – review & editing, Conceptualization. **Alexander Galkin:** Writing – review & editing, Writing – original draft, Visualization, Supervision, Resources, Project administration, Methodology, Investigation, Funding acquisition, Formal analysis, Conceptualization.

## Declaration of competing interest

The authors declare that they have no known competing financial interests or personal relationships that could have appeared to influence the work reported in this paper.

## Data Availability

Data will be made available on request.
