# Corrigendum to “Effect of alternative oxidase (AOX) expression on mouse cerebral mitochondria bioenergetics” [Redox Biol. 77 (2024) 103378]

**DOI:** 10.1016/j.redox.2026.104015

**Published:** 2026-01-13

**Authors:** Belem Yoval-Sánchez, Ivan Guerrero, Fariha Ansari, Zoya Niatsetskaya, Max Siragusa, Jordi Magrane, Vadim Ten, Csaba Konrad, Marten Szibor, Alexander Galkin

**Affiliations:** a Feil Family Brain and Mind Research Institute, Weill Cornell Medicine, 407 East 61st Street, New York, NY, 10065, USA; b Departments of Pediatrics, Robert Wood Johnson Medical School, Rutgers University, New Brunswick, NJ, 08903, USA; c Department of Cardiothoracic Surgery, Center for Sepsis Control and Care (CSCC), Jena University Hospital, Jena, 07747, Germany; d Faculty of Medicine and Health Technology, Tampere University, 33014, Finland

The authors regret that in the originally published article, Fig. 4, panel D contained an incorrect image due to an error during figure file preparation. The correct image for Fig. 4 is shown below.

**Figure:**
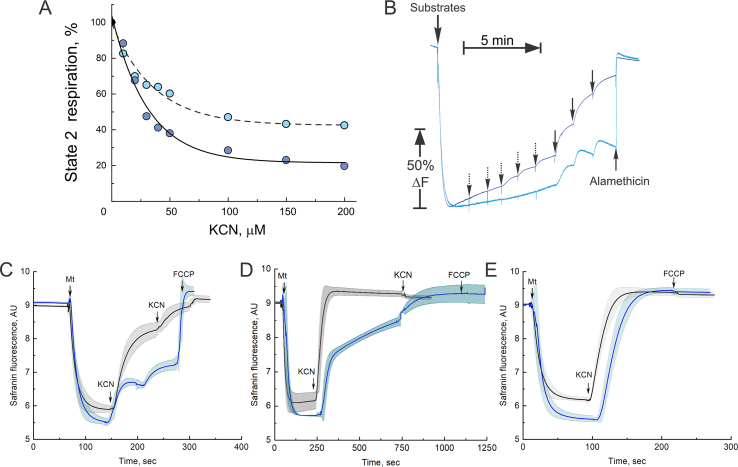

This correction does not affect the results, interpretations, or conclusions of the article. The authors apologize for any confusion this error may have caused.

All authors have reviewed and approved this correction.

The authors would like to apologize for any inconvenience caused.

